# Real-Time Adaptive Respiratory Motion Compensation With Stent-Based Tracking

**DOI:** 10.7759/cureus.84971

**Published:** 2025-05-28

**Authors:** Lee C Goddard, Byung-Han Rhieu, Wolfgang A Tomé

**Affiliations:** 1 Department of Radiation Oncology, Montefiore Medical Center, Bronx, USA; 2 Institute for Onco-Physics, Albert Einstein College of Medicine, Bronx, USA

**Keywords:** adaptive motion management, internal biliary stent, organ-motion tracking, pancreas tumors, radixact synchrony

## Abstract

Stereotactic body radiation therapy (SBRT) offers a promising treatment option for locally advanced pancreatic cancer, but its precision is challenged by respiratory-induced tumor motion. The Radixact Synchrony system integrates real-time motion tracking using internal and external surrogates, potentially enabling reduced treatment margins. In this case study, we report on an 85-year-old female patient with pancreatic cancer who underwent SBRT planning using an implanted biliary stent as a surrogate for tumor motion tracking. Initial assessments, including 4DCT analysis and Synchrony simulation, indicated minimal motion discrepancy between the stent and tumor, supporting its use as a tracking target. However, at the time of treatment, a ~25° rotation of the stent and intermittent external LED signal interruptions impaired motion model accuracy, leading to repeated tracking failures and treatment abortion. The patient was subsequently treated using a free-breathing internal target volume approach. This case highlights both the potential and limitations of using biliary stents as surrogates in real-time motion-adaptive SBRT. It underscores the need for robust quality assurance, improved tracking technologies, and consideration of online adaptive strategies to account for anatomical and surrogate variability. Further investigation is warranted to optimize motion management in pancreatic SBRT utilizing stents as tracking target surrogates.

## Introduction

Stereotactic body radiation therapy (SBRT) is an emerging treatment modality for locally advanced and borderline resectable pancreatic cancer, offering precise dose delivery while minimizing the radiation dose to surrounding healthy tissues [1,2]. Respiratory-induced tumor motion presents a significant challenge, often necessitating motion management strategies to ensure accurate dose deposition [3]. The Radixact Synchrony system (Accuray Inc., Sunnyvale, CA) integrates helical tomotherapy with advanced motion tracking technology, aiming to improve treatment accuracy by adapting to tumor motion throughout treatment delivery [4,5].

The respiratory motion correction model utilizes the onboard planar kV imager and LEDs, which are placed on the patient’s abdomen, to allow for detection of external motion. A model is built before treatment, and images are acquired during treatment, with the model being continually verified and updated with each new kV image. Treatment jaw position and/or MLC openings are offset, based on the model and the external LED position, with the offset being updated every 10 ms. There are two potential respiratory tracking models. The “Fiducial with Respiratory” model detects the position of dense fiducials, with the largest dimension less than 5 mm, which are inserted in or around the tumor. The “Lung with Respiratory” model is designed to track sufficiently dense lesions in the lung. Targets should be contained within a sphere equal to, or less than 8 cm in diameter, with motions less than 2 cm along any translational axis.

Although designed to track dense lesions in the lung, this model can also be employed to track sufficiently dense objects in other parts of the body, such as stents, located within surrounding tissues of higher density than the lung. Endobiliary stents are placed in 70% of newly diagnosed pancreatic cancer patients for decompression of malignant biliary obstruction [6]. Implanted stents within or near the tumor can be used as surrogates for tumor motion tracking, as long as necessary quality assurance steps confirm the co-tracking accuracy of the stent with the target tumor. This approach allows the omission of the burdensome separate fiducial placement procedure, while at the same time enabling precise real-time motion correction, potentially allowing for reduced treatment margins and improved target coverage [7]. However, despite the theoretical benefits, the reliability of stents as tracking surrogates remains uncertain, particularly in the setting of potential intra-fractional stent deformation or rotation.

Here, we present a case of a patient with pancreatic cancer planned for SBRT using the Radixact Synchrony system, where real-time motion correction was successfully modeled based on tracking an implanted stent. However, at the time of treatment, unexpected stent rotation compromised the tracking accuracy, ultimately preventing treatment completion. This case highlights both the potential and the limitations of using implanted stents as motion-tracking surrogates in pancreatic SBRT and underscores the need for further investigation into optimal motion management strategies in this patient population.

## Case presentation

Patient characteristics

**Figure 1:**
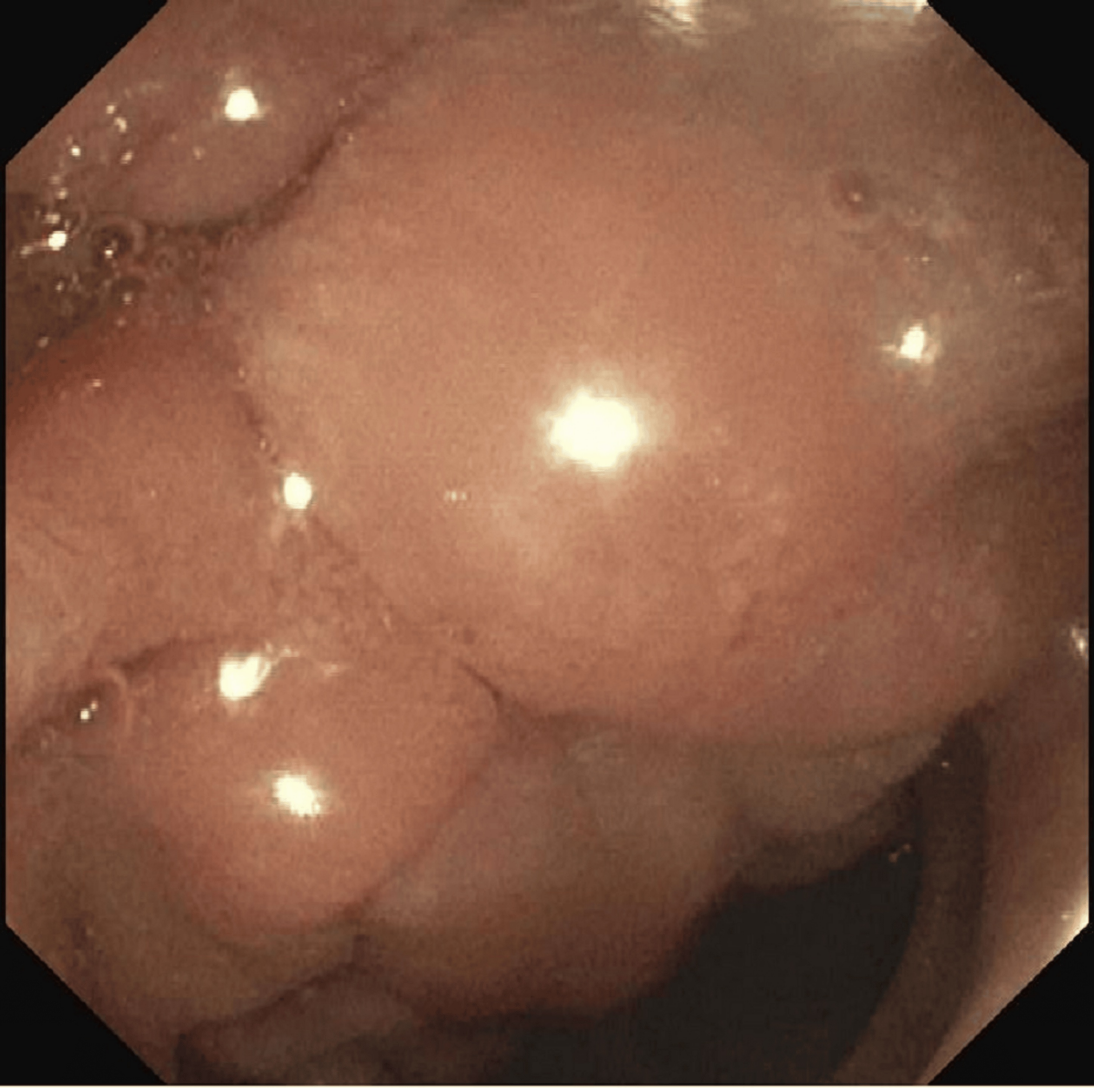
Endoscopic image showing the second portion of duodenum at the level of pancreatic head tumor

**Figure 2:**
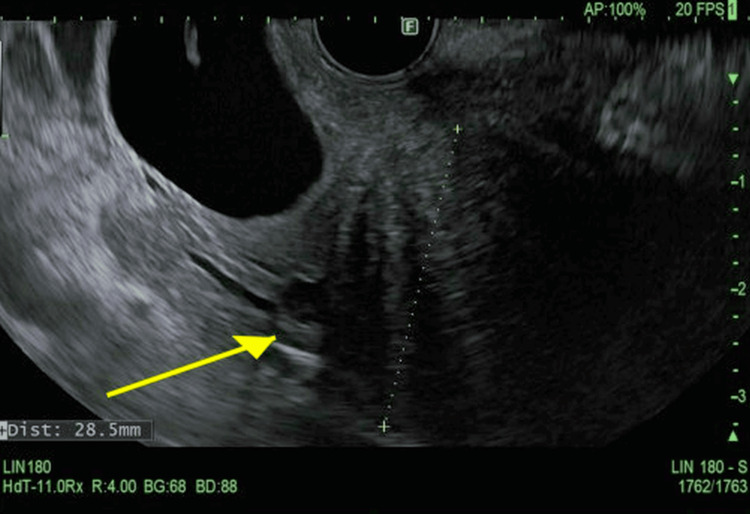
Ultrasound image of the pancreatic mass The dotted line indicates distance measurement of the pancreatic head. The yellow arrow indicates the hypoechoic pancreatic head mass that was targeted with fine needle aspiration

The patient is an 85-year-old woman who was recently diagnosed with locally advanced head of pancreas adenocarcinoma. She initially presented with jaundice and abdominal pain. Imaging showed biliary tract and pancreatic ductal dilatation. Endoscopic ultrasound revealed a pancreatic head mass, as shown in Figures 1, 2, and fine needle biopsy showed adenocarcinoma. She underwent choledochoduodenostomy and placement of a biliary stent (AXIOS™ Stent, Boston Scientific). She was deemed medically inoperable due to her advanced age and limited performance status. For local therapy, she was offered palliative SBRT pending discussion of her systemic therapy. 

CT simulation and treatment planning

**Figure 3:**
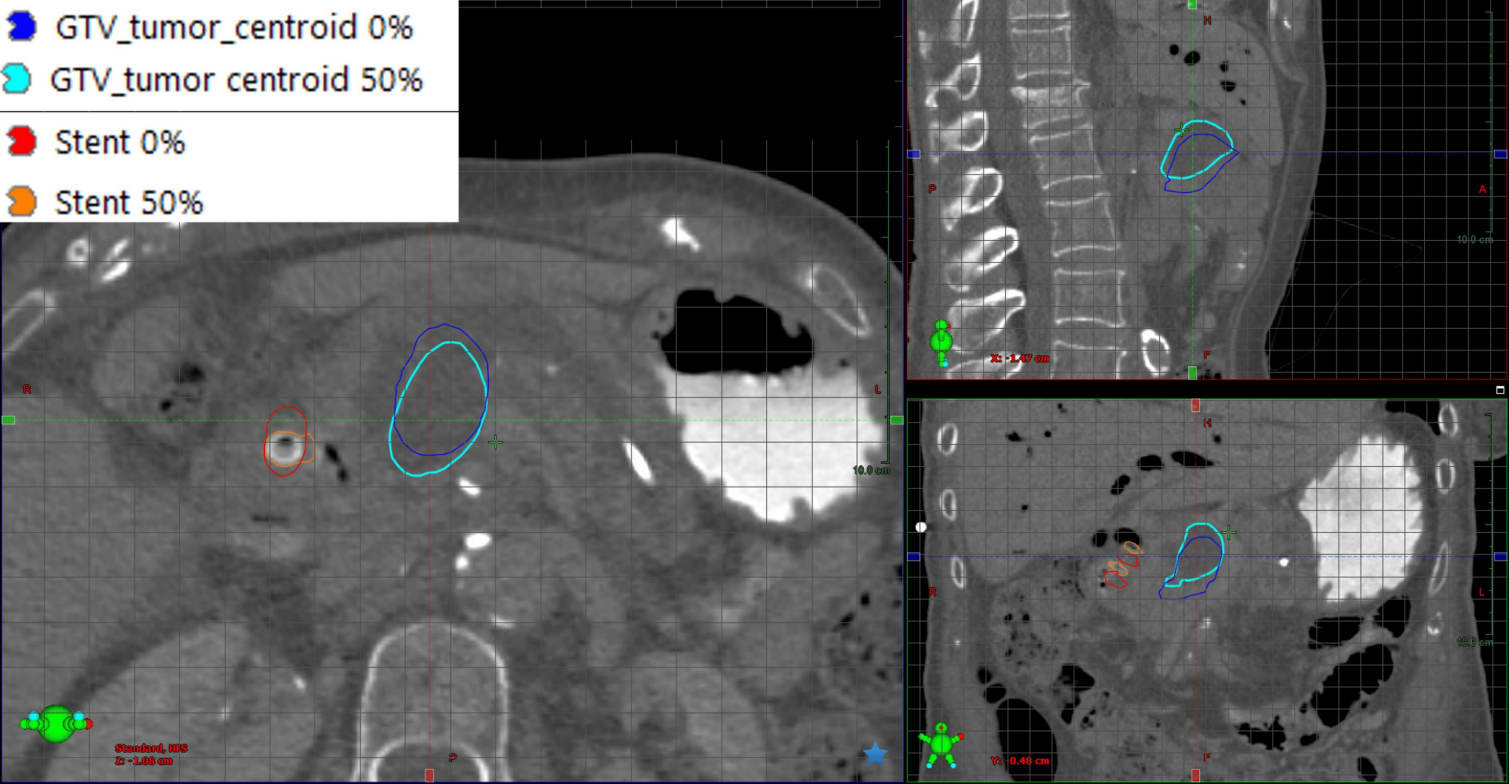
50% phase CT image showing the extrema GTV and stent positions Grid lines show a 1 cm spacing GTV: Gross Tumor Volume

The patient was instructed to withhold food and liquids for four hours prior to CT simulation and treatment. Prior to CT simulation, the patient was immobilized using a BlueBAG™ BodyFIX® T-Shape 1130x1375mm (3C-Medical Intelligence GmbH, Germany), with her arms raised. A free breathing CT was acquired in the head-first supine position utilizing a SOMATOMgo CT Scanner (Siemens Healthineers, Erlangen, Germany) with a 1 mm slice thickness. Breath-hold acquisition was attempted using the Respiratory Gating for Scanners (RGSC) tracking system in combination with audible instructions and a video coaching device (VCD) (Varian, Palo Alto, CA). Unfortunately, the patient was unable to hold her breath repeatedly in a constant amplitude window. The patient then underwent a 4DCT to determine the extent of tumor motion. Ant/Post and Left/Right motion was found to be ~ 1 mm, Sup/Inf motion was found to be ~ 6 mm. A second 4DCT was acquired with oral and IV contrast to allow for improved tumor and organ at risk (OAR) visibility as shown in Figure 3.

The treating physician contoured the gross tumor volume (GTV) on all phases of the contrast 4DCT. A rigid stent abutting the GTV, and calcifications (Cal1 and Cal2) adjacent to the GTV were also contoured using Hounsfield unit thresholding tools. The centroid position of the GTV, stent, and calcifications was calculated on all phases of the 4DCT and the position difference was measured, relative to the stent centroid calculated relative to the 50% phase CT. The GTV centroid was 3.3 cm from the stent centroid, as defined on the 50% phase image. The maximum 3D vector difference in the GTV centroid versus the stent centroid, when analyzed on all other phase images, was found to be 1.4 mm with an average value of 0.8 mm. Similarly, for Cal1, a maximum value of 0.8 mm and an average value of 0.5 mm were found. Cal2 had a maximum deviation of 1.4 mm and an average value of 0.9 mm. The small changes in the relative position of all four structures suggest that the stent could be a good tracking surrogate for GTV motion. The stent volume was found to vary by a maximum of 0.2 cm^3^ between phases, indicating that there were minimal imaging artifacts that could potentially cause deformation of the stent between phases. For normal tissues, GTV and PTV were all contoured on the 50% phase CT image to be used for treatment planning. A 3 mm uniform expansion from the GTV was utilized to create the PTV. The GTV and PTV had volumes of 142.5 cm^3^ and 179.4 cm^3^, respectively. A 4 mm uniform expansion was utilized for normal tissues to generate planning organ at risk volumes (PRVs). Dose evaluation structures were generated by subtracting the PRVs from the GTV and PTV ("GTV_Eval" and "PTV_Eval", respectively).

**Figure 4:**
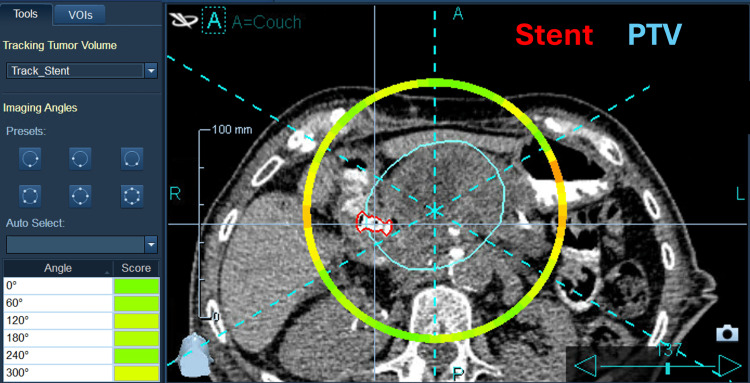
Axial view showing the stent outlined in red and the PTV outlined in cyan. Also shown are the intended imaging angles and the score wheel indicating which angles allow for good target detection PTV: Planning Target Volume

This CT was exported to the Precision treatment planning system (TPS) (Accuray Inc., Sunnyvale, CA). The stent was selected as the tracking target for “Lung with Respiratory” tracking and the TPS determined that the stent should be detectable for the majority of potential imaging angle acquisitions, as shown in Figure 4. The treatment plan utilized the 2.5 cm dynamic jaw and had a gantry period of 12.4s. Six imaging angles were selected, resulting in a kV image acquisition approximately every 2 s. The total treatment time was 399.7 s.

Dose metrics

A total dose of 27.5 Gy was prescribed to the PTV, to be delivered in five fractions. The final treatment plan resulted in 27.5 Gy covering 92.5% of the PTV and 98.3% of the PTV_Eval structures. 27.5 Gy covered 99.6% of the GTV and 100% of the GTV_Eval structures. The PTV conformality index was 1.05, and the homogeneity index was 1.29. The ratio of 50% isodose volume to PTV volume (R50) was calculated to be 5.7. The maximum dose 2 cm away from the PTV was 17.5 Gy or 63.6% of the prescription dose.

Synchrony simulation

Prior to treatment, the patient was brought to the department for a “Synchrony Simulation” appointment. The patient was positioned on the treatment couch utilizing the same immobilization device that was utilized at CT simulation. Three LED markers were placed on the patient’s abdomen by the treating therapists at the position of the largest motion amplitude. A fourth marker was also placed on the treatment couch.

**Figure 5:**
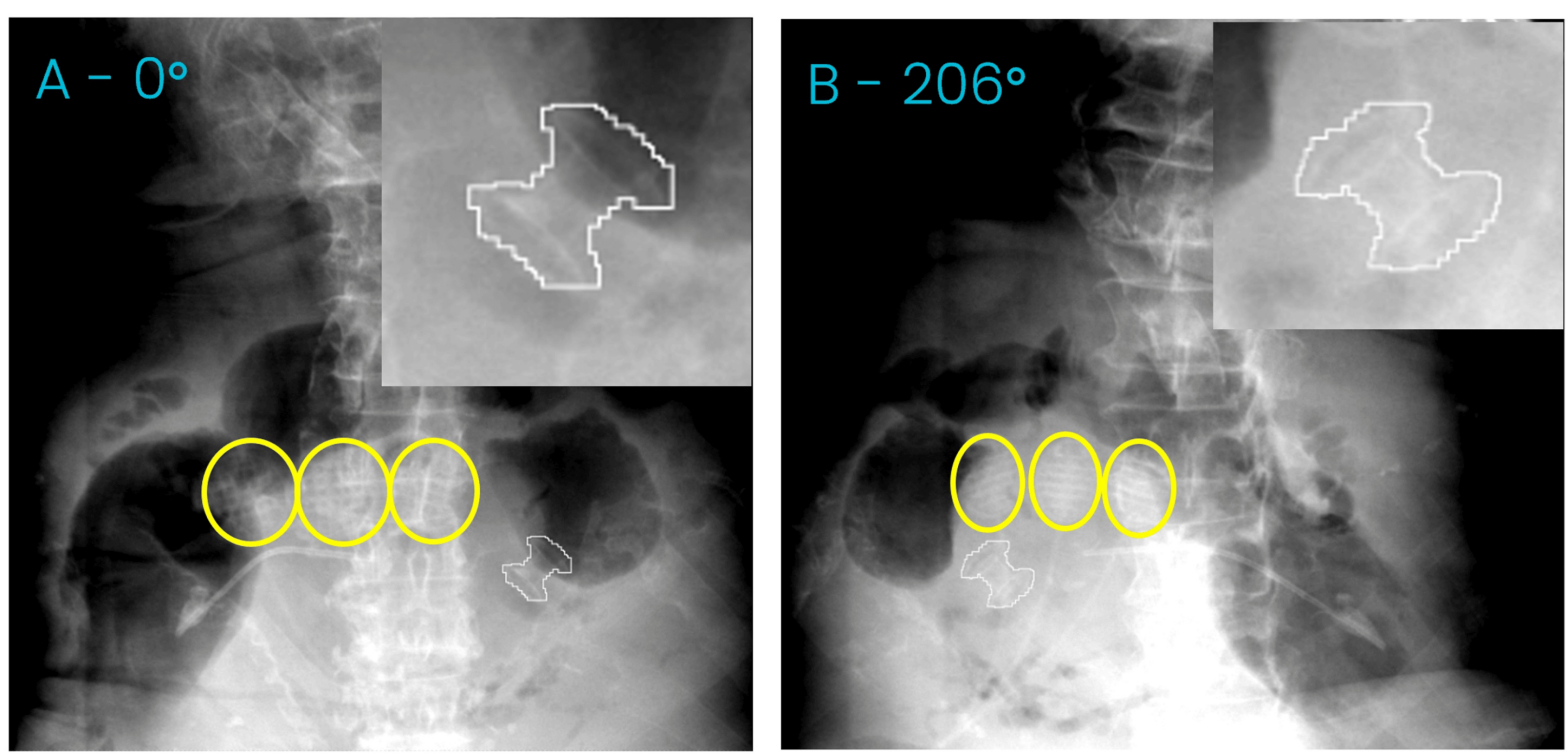
Two acquired kV images from the simulation session with gantry angle A) 0 degrees and B) 206 degrees. The expected stent position is shown outlined in white and enlarged. Yellow circles show the LED markers

During this appointment, optimal imaging angles were determined, kV acquisition parameters were set, and a model of the internal target motion versus external LED motion was built. Once a model is built, the treatment couch and gantry move at the same speed as in the treatment plan; however, no treatment beam is enabled. New kV images are acquired as the gantry rotates around the patient, and the model is verified and updated with each new image. During this process, there were two interruptions, causing the model to pause treatment. At each interruption, a new model could be successfully built, and the simulated treatment could be resumed. Figure 5 shows two of the acquired images with the detected stent position overlayed in white and enlarged. Yellow circles indicate the position of the LED markers placed on the patient's abdomen.

Treatment delivery

After the successful simulation appointment, the patient was brought back to the department the following day for treatment. Pre-treatment kVCT imaging was acquired, and it was noted that the position of the stent was shifted. Model imaging acquisition was undertaken, and a model was unable to be successfully built when utilizing the imaging angles from the simulation appointment. The imaging angles were adjusted, and a model was successfully built; however, upon initiating treatment, the model would only successfully treat for a short period of time before having to be rebuilt. It was noted at this time that LED3 was having frequent interruptions. The treating therapist adjusted the LED position and found that the patient was asleep. Treatment was once again initiated, but after a number of model interruptions, it was decided to halt the treatment for the day and to complete the remaining treatments utilizing an internal target volume (ITV) and free-breathing technique. A repeat 4DCT was acquired immediately following the attempted treatment fraction to be utilized to generate the new free-breathing treatment plan.

**Figure 6:**
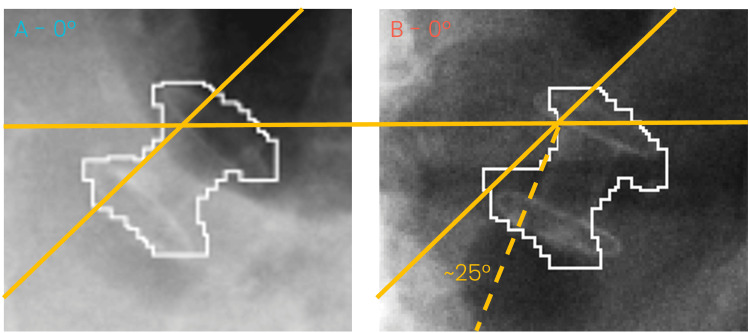
kV planar images at gantry 0 degrees for A) the simulation appointment and B) attempted treatment. As shown in B, the stent is rotated approximately 25 degrees from the expected position, shown in white

**Figure 7:**
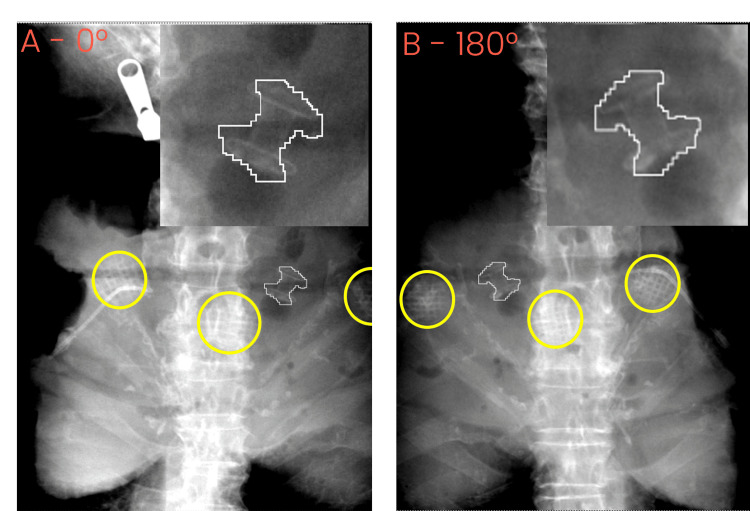
Two acquired kV images from the attempted treatment with gantry angle A) 0 degrees and B) 180 degrees. The expected stent position is shown outlined in white and enlarged. Yellow circles show the LED markers

After the aborted treatment, the data from both the simulation treatment and first treatment were analyzed with the help of Accuray’s physics team. Two crucial issues with the treatment fraction were noted during the analysis. The primary issue was the stent position, which was noted to have rotated by ~ 25 degrees, when viewed in the anterior plane, as shown in Figure 6. Figure 7 shows the same rotation at both the gantry 0 and gantry 180 positions. The second issue was caused by the intermittent LED3 connection, resulting in fewer available LED positions to allow for a model to be successfully built.

## Discussion

Since two-thirds of pancreatic cancer patients undergo biliary stent placement at their initial presentation for decompression of malignant biliary obstruction, a biliary stent can be an appealing tumor tracking surrogate marker in place of dedicated fiducials. A separate procedure for fiducial placement is burdensome for these patients and leads to an extra step that delays SBRT treatment initiation. There are known caveats with biliary stent tracking, including stent migration and deformation. The rate of biliary stent migration has been reported to be approximately 5-10% [8]. Prior to treatment, analysis of the acquired 4DCT showed minimal positional variation between the stent and tumor centroid across respiratory phases. When exported to the TPS, the stent was found to be sufficiently dense, in comparison to the surrounding tissue, that it could be utilized as a tracking target. These findings suggested that the stent could be employed for real-time motion adaptation potentially allowing the use of reduced treatment margins. During the Synchrony simulation, the system was able to verify and update the model throughout the simulated treatment, further supporting the feasibility of this tracking approach. However, during actual treatment delivery, significant challenges arose. The primary issue was an unexpected ~25-degree rotation of the stent in the anterior plane. This rotational movement compromised the accuracy of tumor tracking, leading to frequent interruptions and the inability to complete treatment as planned. Unlike small translational deviations, which can often be corrected by real-time model updates, rotational changes in the stent orientation directly impacted the system’s ability to detect the stent on kV planar imaging. This highlights a critical limitation of using implanted stents as tracking targets in the current implementation of the Synchrony real-time tracking system, as their stability is not guaranteed over time and may be influenced by patient anatomy, peristalsis, or other physiological factors.

In addition to the stent rotation, intermittent connection issues with one of the LED motion markers contributed to modeling failures. The external LEDs serve as a reference for correlating internal tumor motion with respiratory patterns, and any loss of LED signal integrity can result in inadequate motion model generation. In this case, the LED connection issue led to further interruptions, reducing the effectiveness of the Synchrony real-time target tracking system. These findings emphasize the importance of robust external motion tracking and suggest that additional quality control measures should be implemented to ensure reliable LED function throughout treatment.

Following this unsuccessful attempt at motion-adaptive SBRT, the treatment strategy was modified to a free-breathing technique with an ITV approach. This alternative method, which accounts for tumor motion by expanding the target volume rather than actively adapting to real-time movement, allowed for the successful completion of SBRT. While this approach reduces reliance on motion tracking, it necessitates larger margins, potentially increasing radiation exposure to adjacent organs at risk.

This case highlights the need for further investigation into the optimal motion management strategies for pancreatic SBRT. While the use of stents as motion-tracking surrogates shows promise, rotational stability must be thoroughly evaluated before implementation in clinical practice. Alternative strategies, such as the implantation of dedicated fiducial markers, hybrid tracking techniques, and online adaptive planning approaches, may improve treatment reliability. Additionally, further refinement of the Synchrony system’s ability to detect and compensate for rotational motion could enhance its applicability in pancreatic cancer treatment.

Ultimately, this case underscores the complexities of real-time motion tracking in pancreatic SBRT. While advanced motion management systems like Radixact Synchrony hold exciting potential for improving treatment precision, technical challenges such as surrogate stability and tracking reliability must be carefully addressed to optimize clinical outcomes. Future studies should focus on refining patient selection criteria, improving tracking algorithms, and exploring alternative fiducial markers to enhance the robustness of motion-adaptive SBRT in pancreatic cancer.

## Conclusions

This case highlights the limitations of using implanted biliary stents as motion-tracking surrogates in pancreatic SBRT, particularly due to potential rotational instability. To prevent similar issues, in patients who are unable to perform breath-hold or other motion-limiting strategies, online adaptive planning could be utilized to contour tracking targets on the daily kVCT image, ensuring real-time adjustments based on their actual position and orientation.

Beyond improving motion tracking, online adaptive planning also offers the advantage of accounting for daily variations in OAR anatomy and tracking targets and their positional relation to the GTV. Changes in bowel gas, stomach filling, or other anatomical shifts can significantly impact the dose distribution. By adapting the treatment plan to the daily kVCT, radiation delivery can be optimized to maintain target coverage while minimizing OAR exposure. Integrating these adaptive strategies with real-time motion management may enhance the precision and reliability of SBRT for pancreatic cancer, ultimately improving treatment outcomes.
